# Characterization of *meta*-Cresol Purple for spectrophotometric pH measurements in saline and hypersaline media at sub-zero temperatures

**DOI:** 10.1038/s41598-017-02624-0

**Published:** 2017-05-30

**Authors:** Socratis Loucaides, Victoire M. C. Rèrolle, Stathys Papadimitriou, Hilary Kennedy, Matthew C. Mowlem, Andrew G. Dickson, Martha Gledhill, Eric P. Achterberg

**Affiliations:** 1 University of Southampton, Waterfront Campus, Southampton, SO14 3ZH UK; 2 National Oceanography Centre, European Way, Southampton, SO14 3ZH UK; 3 Ocean Sciences, College of Natural Sciences, Bangor University, Menai Bridge, LL59 5AB UK; 4 Marine Physical Laboratory, Scripps Institution of Oceanography, University of California, San Diego, 9500 Gilman Drive, La Jolla, CA 92093-0244 USA; 5 GEOMAR Helmholtz Centre for Ocean Research, 24148 Kiel, Germany

## Abstract

Accurate pH measurements in polar waters and sea ice brines require pH indicator dyes characterized at near-zero and below-zero temperatures and high salinities. We present experimentally determined physical and chemical characteristics of purified meta-Cresol Purple (*m*CP) pH indicator dye suitable for pH measurements in seawater and conservative seawater-derived brines at salinities (S) between 35 and 100 and temperatures (T) between their freezing point and 298.15 K (25 °C). Within this temperature and salinity range, using purified *m*CP and a novel thermostated spectrophotometric device, the pH on the total scale (pH_T_) can be calculated from direct measurements of the absorbance ratio R of the dye in natural samples as

$${\boldsymbol{p}}{{\boldsymbol{H}}}_{{\boldsymbol{T}}}{\boldsymbol{=}}{\boldsymbol{-}}{\bf{log}}({{\boldsymbol{k}}}_{{\bf{2}}}^{{\boldsymbol{T}}}{{\boldsymbol{e}}}_{{\bf{2}}}){\boldsymbol{+}}\,{\bf{log}}(\frac{{\boldsymbol{R}}{\boldsymbol{-}}{{\boldsymbol{e}}}_{{\bf{1}}}}{{\bf{1}}{\boldsymbol{-}}{\boldsymbol{R}}\frac{{{\boldsymbol{e}}}_{{\bf{3}}}}{{{\boldsymbol{e}}}_{{\bf{2}}}}})$$

Based on the *m*CP characterization in these extended conditions, the temperature and salinity dependence of the molar absorptivity ratios and − ${\bf{log}}({{\boldsymbol{k}}}_{{\bf{2}}}^{{\boldsymbol{T}}}{{\boldsymbol{e}}}_{{\bf{2}}})$ of purified *m*CP is described by the following functions: *e*_1_ = −0.004363 + 3.598 × 10^−5^ *T*, *e*_3_/*e*_2_ = −0.016224 + 2.42851 × 10^−4^ *T* + 5.05663 × 10^−5^(*S* − 35), and − ${\bf{log}}({{\boldsymbol{k}}}_{{\bf{2}}}^{{\boldsymbol{T}}}{{\boldsymbol{e}}}_{{\bf{2}}})$ = −319.8369 + 0.688159 *S* −0.00018374 *S* ^2^ + (10508.724 − 32.9599 *S* + 0.059082*S* ^2^) T^−1^ + (55.54253 − 0.101639 *S*) ln T −0.08112151*T*. This work takes the characterisation of *m*CP beyond the currently available ranges of 278.15 K ≤ T ≤ 308.15 K and 20 ≤ S ≤ 40 in natural seawater, thereby allowing high quality pH_T_ measurements in polar systems.

## Introduction

About half of the anthropogenic carbon dioxide (CO_2_) released to the atmosphere since the industrial revolution has been absorbed by the oceans^1^. This process continues today and buffers atmospheric CO_2_ levels, thereby partly alleviating global warming. The influx of CO_2_ into the ocean causes acidification of surface waters and leads to a decline in the saturation states of carbonate minerals (i.e. aragonite and calcite), posing a threat to marine calcifying species^2–4^. The capacity of ocean waters to absorb CO_2_ increases towards the poles because of the higher solubility of gasses at lower temperatures^5^. High freshwater inputs into polar waters, from ice and snow melt, reduce the seawater’s buffering capacity, as indicated by the Revelle factor^6^, leading to a decline in pH and saturation states of calcite and aragonite^7, 8^. The contemporary ocean shows the lowest buffering capacity (highest Revelle factor) in polar waters^9^, and it is projected that by the end of the century these regions will become undersaturated with respect to aragonite^10, 11^.

Although high latitude waters contribute disproportionally to the oceanic CO_2_ uptake^5, 12^, the flux estimates are based on data available from periods of seasonal sea ice retreat and parts of the ocean which are ice-free^13^. Over the last few years the role of sea ice processes in CO_2_ cycling has been increasingly recognised. Sea ice is a porous medium and within its pores and channels are gas pockets and residual high ionic strength liquids (brines) at thermal equilibrium with the ice^14^. The brine, enriched in seawater solutes rejected from the ice during freezing^14^, is the habitat of sympagic phototrophic and heterotrophic organisms^15, 16^. It has been estimated that in first- and multi-year ice packs of the Southern Ocean, primary production results in the fixation of 36 Tg C yr^−1^ into biomass^17^. It is now accepted that the sea ice pack and land fast ice are to a measurable extent CO_2_ permeable and that internal physical, chemical, and biological processes taking place during ice formation and melting may play a significant role in CO_2_ cycling in high latitude oceans^18–20^. For example, gravity drainage of CO_2_-rich brines during ice formation may be a significant and so far unaccounted sink of dissolved inorganic carbon (DIC) in surface waters with estimates in the order of 200–500 Tg C yr^−1^ for the (Arctic and Antarctic) polar oceans^21^. Carbonate mineral precipitation in brines during ice formation may present a potentially significant source of total alkalinity (TA) to polar surface waters following their dissolution when sea ice melts, generating an additional sink (~33–83 Tg C yr^−1^) of atmospheric CO_2_, which is equivalent to 17–42% of the air-sea CO_2_ flux in open high latitude ocean waters^22^. In addition to these mechanisms (gravity drainage, CaCO_3_ formation in sea ice), based on recent direct measurements of the CO_2_ exchange between sea ice and the atmosphere as a function of ice temperature, the Antarctic ice pack, during seasonal warming, was estimated to take up the equivalent of 58% of the atmospheric CO_2_ uptake of the open Southern Ocean surface waters south of 50°S^23^. The interplay between biological and physicochemical processes makes carbonate chemistry within sea ice highly complex, leading to strong gradients in pH between the ice and underlying waters with potentially significant impacts on ocean-atmosphere CO_2_ fluxes^15, 18, 24–26^.

Our ability to characterize the marine carbonate system in open ocean waters has undergone major advancements during the last few decades, but our understanding of CO_2_ cycling in ice brine conditions remains limited due to theoretical and methodological constraints^25^. Sea ice brines exhibit a much wider range of salinity (S) and temperature (T) changes within short temporal and spatial scales than the open ocean. Specifically, brine S-T conditions in sea ice extend to the hypersaline region (S > 100) at temperatures much colder than the freezing temperatures of seawater (271.23 K at S = 35 and 0 dbar pressure)^18, 20, 27^. Such large ranges in T and S make the use of traditional *ex situ* pH and *p*CO_2_ (partial pressure of CO_2_) measurement techniques a challenge, because *in situ* temperature corrections are required post-analysis using relationships and constants that have not been validated for below- zero temperatures. The most robust method for back-calculating pH and *p*CO_2_ to *in situ* T relies on the solution of a thermodynamic model that describes the marine CO_2_ system^28^. This requires the knowledge of the first and second acidity constant of carbonic acid at *in situ* T and S. Empirical data for these constants, however, are not available to date for T < 274.15 K and S > 50 in natural seawater while non-linear extrapolation to low T and high S can potentially result in large errors in calculated pH and *p*CO_2_ values^29^.

Experimental determination of the carbonic acid acidity constants can be facilitated by measurements of all four variables (DIC, TA, pH, *p*CO_2_) of the marine carbonate system at the S and T of interest. Although measurements of TA, DIC, and *p*CO_2_ at sub-zero temperatures and hyper-saline conditions are possible using current methodologies and instrumentation^28^, spectrophotometric pH measurements are limited to the range of conditions for which indicators have been characterised. For example, the characterization of the commonly used indicator dye meta-Cresol Purple (*m*CP) is only valid for 278.15 K ≤ T ≤ 308.15 K and 20 ≤ S ≤ 40^30, 31^. Furthermore, pH measurements at low temperatures using conventional optical apparatus (spectrophotometers, glass cells, lenses etc.) are highly problematic due to the formation of condensation along the optical path.

The purpose of this work was to facilitate pH measurements in cold and hypersaline conditions, such as those encountered in the oceanic cryosphere. To this end, we extended the characterization of the pH indicator *m*CP (in its purified form) to below-zero temperatures down to the freezing point (267.15 K) of S = 100 brines. The salinity maximum and temperature minimum were set by the S-T range in natural sea ice brines with conservative ionic composition and inter-ionic ratios relative to surface oceanic water. This development became possible by the recent electrochemical characterization of the pH of the Tris/HCl buffer system^32^ and the use of a novel, custom-made microfluidic spectrophotometric system. The lens-less design of the microfluidic chip prevents condensation and is thus ideal for pH measurements at a lower range of temperatures. Our work facilitates high quality *in-situ* measurements of pH, thereby furthering our understanding of the carbonate system in polar aquatic environments.

## Methods

### Purification of meta-Cresol Purple

The *m*CP indicator dye was obtained as a sodium salt (Acros Organics). The indicator was purified using the preparative HPLC procedure described in Liu *et al*.^31^ using a Shimadzu HPLC system. In preparative mode, the system consisted of a system controller (SCL-10Avp), a preparative scale pump (LC-8A), a Rheodyne 3725i manual injector, and a diode array detector (SPD M10Avp) with a preparative flow cell. In analytical mode, the preparative pump was replaced with an analytical scale pump (LC-10ADvp) and the manual injector with an automatic injector (SIL 10AD). The HPLC column (Primesep B2) used for the purification of *m*CP was from SIELC Technologies. The Primesep B2 column uses a mixed-mode resin to separate analytes via ion-exchange and hydrophobic mechanisms. A preparative column (Part B2–220.250.0510, 22 × 250 mm, particle size 5 μm) was used for the purification procedure while a smaller analytical column (Part B2-46-250.0510, 4.6 × 250 mm, particle size 5 μm) was used for the qualitative analysis of the purified indicator.

The mobile phase used for the purification was 70% acetonitrile (HPLC grade; Fisher Chemical) and 30% deionised water (Milli-Q, Millipore, MQW). A small amount (0.05%) of trifluoroacetic acid (TFA; ReagentPlus^®^; Sigma-Aldrich) was used as a mobile phase modifier. The un-purified *m*CP sodium salt was dissolved in the mobile phase at a concentration of 70 mM. The solution was sonicated in an ultrasonic bath for 15 min to ensure complete dissolution of the indicator. For each purification cycle, 7 mL of indicator solution was injected into the system. The pump flow rate was adjusted to 31 mL min^−1^ and the pure *m*CP was collected at its characteristic retention time (approximately 20 min). The pure *m*CP was separated from the solvent using a rotary evaporator at 40 °C under partial vacuum. Complete evaporation of the mobile phase was achieved after 2–3 h and the recovery efficiency was about 60%. The purified *m*CP (in acid form) was collected from the evaporation flask and its purity was tested using an analytical HPLC procedure. This was done by injecting 0.020 mL of 70 mM purified *m*CP (in mobile phase) through the analytical HPLC system at a flow rate of 1.5 mL min^−1^. The *m*CP purity was assessed by comparing the chromatographs of the purified and unpurified material.

**Figure 1:**
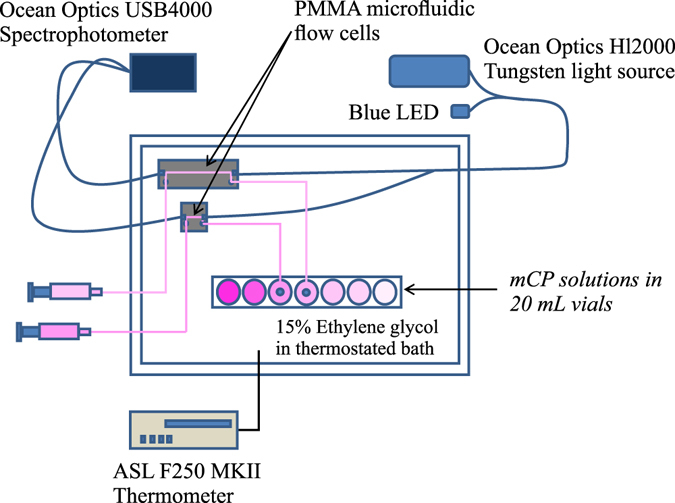
Experimental setup used for the determination of the molar absorptivity ratios e_1_ and e_3_/e_2_. The microfluidic flow cells and vials with *m*CP solutions are submerged in a 15% ethylene glycol thermostated bath. The light is transmitted from the light source to the flow cells and to the spectrophotometer through 600 µm diameter optical fibres (Thorlabs, USA).

### Characterization Procedure

Sulfonephthaleine pH indicator dyes are weak acids (H_2_I) where the acidic and basic components exhibit different colours and, therefore, absorb light at distinctly different wavelengths. For *m*CP, H_2_I is pink, HI^−^ is yellow and I^−2^ is purple. The relative distribution of the indicator species is pH-dependent and can be expressed in terms of chemical equilibria with corresponding dissociation constants:

1 $$H{I}^{-}+{H}^{+}\iff {H}_{2}I\,\,\,{K}_{1}^{T}=\frac{[{H}_{2}I]}{[{H}^{+}][H{I}^{-}]},$$

2 $${I}^{2-}+{H}^{+}\iff H{I}^{-}\,\,\,{K}_{2}^{T}=\frac{[H{I}^{-}]}{[{H}^{+}][{I}^{2-}]},$$

where brackets denote concentration. At typical surface seawater pH (~8.1), *m*CP is present only as I^−2^ and HI^−^ because p*K*_1_^T^ ~2 and p*K*_2_^T^~ 8. At a sample pH close to the log of the indicator’s second dissociation constant (p*K*_2_^T^), pH can be measured with considerable accuracy (better than 0.001) by measuring light absorption at the wavelengths of maximum absorbance of the acidic (HI^−^) and basic (I^−^) indicator species (434 and 578 nm, respectively).

Measurements of pH using indicator dyes require that their optical properties are carefully characterized. The characterization of *m*CP involves the determination under different T and S conditions of the molar absorptivity constants (ε^i^_λ_) of each indicator species (i) at wavelengths (λ) of 434 and 578 nm and the second dissociation constant *K*_2_^T^ (equation 2). Solution pH can then be calculated from the absorbance (*A*_*λ*_) ratio at 434 and 578 nm ($R=\frac{{A}_{578}}{{A}_{434}}$) using:

3 $$p{H}_{T}=-\mathrm{log}({k}_{2}^{T})+\,\mathrm{log}(\frac{R-{e}_{1}}{{e}_{2}-R{e}_{3}})$$

where the parameters e_1_, e_2_ and e_3_ are the molar absorptivity ratios defined by:

4 $${e}_{1}=\frac{{\varepsilon }_{578}^{H{I}^{-}}}{{\varepsilon }_{434}^{H{I}^{-}}};{e}_{2}=\frac{{\varepsilon }_{578}^{{I}^{2-}}}{{\varepsilon }_{434}^{H{I}^{-}}};{e}_{3}=\frac{{\varepsilon }_{434}^{{I}^{2-}}}{{\varepsilon }_{434}^{H{I}^{-}}}$$

The derivation of equation 3 is described in detail in Zhang and Byrne^33^.

Equation 3 can be rearranged to ref. 31:

5 $$p{H}_{T}=-\mathrm{log}({k}_{2}^{T}{e}_{2})+\,\mathrm{log}(\frac{R-{e}_{1}}{1-R\frac{{e}_{3}}{{e}_{2}}})$$

which simplifies the characterization procedure since e_3_/e_2_ is determined as a single parameter in a basic solution (pH ~12) where I^2−^ is the predominant indicator species so that:

6 $$\frac{{e}_{3}}{{e}_{2}}=\frac{{\varepsilon }_{434}^{{I}^{2-}}}{{\varepsilon }_{578}^{{I}^{2-}}}$$

Applying Beer-Lambert’s law, and as long as ${\varepsilon }_{434}^{{I}^{2-}}$ and ${\varepsilon }_{578}^{{I}^{2-}}$ are measured in the same solutions, e_3_/e_2_ simply becomes the ratio between ${A}_{434}^{{I}^{2-}}$ and ${A}_{578}^{{I}^{2-}}$ eliminating the need for precise knowledge of the concentration of *m*CP. This, however, presents its own challenge since the absorbance of I^2−^ at 578 nm is much higher than at 434 nm making it difficult to determine both absorbances accurately from a single measurement. To overcome this, we measured the absorbances of the same solutions in two different absorption cells: 1-cm-path length for ${A}_{578}^{{I}^{2-}}$ and a 10-cm-path length for ${A}_{434}^{{I}^{2-}}$. This ensured that absorption measurements of both *m*CP species were within acceptable ranges and eliminated errors associated with *m*CP dilution preparation uncertainties. Maximum errors in the length of each absorption cell were 5 µm which translates to a maximum error of 0.045% in e_1_ or e_3_/e_2_ and of 0.00002 in pH.

Absorption measurements for the determination of e_3_/e_2_ were made in *m*CP solutions with ionic composition similar to that of seawater and pH adjusted to ~12 with 1 M NaOH. To avoid precipitation of magnesium, sulphur and carbonate salts at high pH and salinities, MgCl_2_ was replaced with CaCl_2_ and Na_2_SO_4_ and NaHCO_3_ with NaCl. The ionic strength of the solutions was adjusted accordingly to match that of seawater and brines up to S = 110. The e_3_/e_2_ was determined by measuring A_434_ and A_578_ in a series (n = 6–10) of *m*CP dilutions from 5–50 µM concentration.

We followed the same approach as described above for the determination of e_1_, using the 1 cm cell to determine ${A}_{434}^{H{I}^{-}}$ and the 10 cm cell for ${A}_{578}^{H{I}^{-}}$. Absorbance measurements were made at *m*CP concentrations between 10 and 600 µM (n = 6–10) in NaCl solutions buffered with 0.02 M CH_3_COONa with ionic strength equivalent to that of seawater and brines up to S = 110. The pH of these solutions was adjusted to 4.5 by addition of small amounts of 1 M HCl. The maximum salinity used for the determination of e_1_ and e_3_/e_2_ (S = 110) brackets the maximum salinity at which the pH_T_ of the Tris/HCl buffers (S = 100) has been determined^32^ (see below). The latter salinity sets the upper limit of the salinity range for the $-\mathrm{log}({k}_{2}^{T}{e}_{2})$ determined in this study.

The molar extinction coefficients (${\varepsilon }_{434}^{{I}^{2-}}$, ${\varepsilon }_{578}^{{I}^{2-}},{\varepsilon }_{434}^{H{I}^{-}}$ and ${\varepsilon }_{578}^{I{H}^{-}}$) were determined using the Beer-Lambert Law rearranged to ${\varepsilon }_{\lambda }^{i}=\frac{{A}_{\lambda }}{b\times {C}_{mCP}}=\frac{a}{b}$, where *a* is the slope of the linear regression of absorbances versus concentrations of the *m*CP dilution series and *b* is the length of the optical cell. Although molar extinction coefficients have been traditionally determined through repeat absorption measurements of a single *m*CP concentration (as in a single point calibration) we have opted for a multi-point regression approach to establish the linear range of our measurements and to account for intercept offsets.

The $-\mathrm{log}({k}_{2}^{T}{e}_{2})$ term in equation 5 was determined by the measurement of the absorbance ratio $R=\frac{{A}_{578}}{{A}_{434}}$ in Tris/HCl buffers in synthetic seawater and synthetic seawater-derived brines (S = 35–100). The buffers were prepared and their pH was characterized electrochemically on the total proton scale (pH_T_) in the 267.15 K to 298.15 K temperature range with the Harned cell at the Marine Physical Laboratory, Scripps Institution of Oceanography, University of California San Diego^32^. The equimolal Tris/HCl buffer (0.08 m Tris, 0.04 m HCl) has been previously used for this purpose^31^, and the salinity and temperature dependence of its pH_T_ in the current, extended S–T range has been determined [equimolal Tris/HCl: pH_T_ = 536.08338–54.732367 *S* + 0.8518518 *S* ^2^ + (0.1675218−1.72224095 × 10^−2^ *S* + 2.66720246 × 10^−4^ *S* ^2^) T + (−10873.5234 + 1369.56485 *S*−21.34442 *S* ^2^) T^−1^ + (−95.04342 + 9.7014355 *S*–0.1509014 *S* ^2^) lnT (standard error: 0.001 pH unit)]^32^. However, this buffer was increasingly basic at low temperatures and high salinities (e.g., pH_T_ = 8.09 at T = 298.15 K and S = 35; pH_T_ = 9.19 at T = 269.15 K and S = 70)^32^. So, two sets of less alkaline buffers, each set with distinctly different non-equimolal Tris/HCl composition (0.06 m Tris, 0.04 m HCl; and 0.10 m Tris, 0.06 m HCl) were prepared and used for the determination of $-\mathrm{log}({k}_{2}^{T}{e}_{2})$ at S = 35–100. The (0.06 m Tris, 0.04 m HCl) buffers s were characterized electrochemically at Scripps^32^ and used for the *m*CP characterization experiments at S = 35, 45, 50, 60, 70, 85, and 100. Their pH_T_ was calculated from the reported best-fit function, pH_T_ = 144.4361–1.0809685 *S* + 0.006023772 *S* ^2^ + (0.0618411−0.000817397 *S* + 4.27187 × 10^−6^ *S* ^2^) T + (−27.233738 + 0.2329236 *S*–0.001281138 *S* ^2^) lnT, with a standard error of 0.002 pH unit^32^. The (0.10 m Tris, 0.06 m HCl) buffers were used for additional *m*CP characterization experiments at S = 35 and 45. The pH_T_ of the (0.10 m Tris, 0.06 m HCl) buffers was not characterized electrochemically (except for the S = 45 buffer at 273.15 K, see below) but instead computed from the equimolal pH_T_ (as calculated from the best-fit equation cited above) via the Henderson–Hasselbalch equation^32, 34^. This computation gives pH_T_ = 8.785 at 273.15 K for the S = 45 (0.10 m Tris, 0.06 m HCl) buffer, which agrees well with the value determined electrochemically (pH_T_ = 8.783) as described in Papadimitriou *et al*.^32^. This approach is also supported from the excellent agreement between thus computed and electrochemically determined pH_T_ values for the (0.06 m Tris, 0.04 m HCl) buffers^32^.

### Spectrophotometric measurements

The experimental set-up used for the determination of molar absorptivity constants (ε^i^_λ_) is illustrated in Fig. 1. The microfluidic flow cells used for the characterization were manufactured in tinted poly (methyl methacrylate) (PMMA). The fabrication procedure is described in detail in Ogilvie *et al*.^35^ and Floquet *et al*.^36^. Two absorption cells (1 cm and 10 cm) with cross sections of 700 µm × 700 µm were micro-milled into a single PMMA chip. A tungsten halogen light source (Ocean Optics HL-2000) was used for the absorption measurements in conjunction with a 434 nm LED used to boost light intensity at the lower end of the spectrum. A linear array photodiode spectrophotometer (USB4000, Ocean Optics, UK) was used as a detector. Both the light source and detector were connected to the microfluidic flow cell with 600 µm diameter optical fibres (Thorlabs, USA). The flow cell was submerged in a water bath (Grant TX150) filled with 15% ethylene glycol solution. The temperature was kept constant (±0.02 °C) and was monitored continuously using a precision thermometer (ASL F250 MKII). The lens-less design of the PMMA microfluidic flow cell allowed for uncompromised optical measurements of pH (no condensation issues) and superior thermostatic control at near-freezing temperatures.

For the determination of the molar absorptivity constants (ε^i^_λ_), experimental solutions were volumetrically premixed with *m*CP indicator using calibrated pipettes in 20 mL glass vials with silicone/PTFE septum tops. The vials were kept on a rack which was submerged in the water bath. Solutions were siphoned from the vials through a 0.7 mm i.d. PTFE capillary tube into the flow cell using a 1 mL disposable syringe connected to the outlet of the flow cell. The flow cell was flushed with 2 mL of the experimental solution between measurements. The absorption spectrum was recorded in replicate (n = 5) using LabVIEW® software. Reference measurements were performed in experimental solutions without added indicator.

For the determination of $-\mathrm{log}\,{k}_{2}^{T}{e}_{2}$, the $R=\frac{{A}_{578}}{{A}_{434}}$ was determined inTris/HCl buffers using the microfluidic pH sensor as described in Rérolle *et al*.^37^ but with the same spectrophotometer and light source described above. For each measurement, 4 µL of the 4 mM *m*CP solution was mixed with 900 µL Tris/HCl buffer. The impact of the *m*CP addition on the buffer pH was estimated by measuring pH over a wide range of *m*CP to buffer mixing ratios (1:25 to 1:80) and using this data to regress back to a theoretical pH where *m*CP concentration was zero. This range of mixing ratios was obtained from the dispersion of *m*CP in Tris/HCl buffer within the microfluidic channels^37^. The measurements for the determination of $-\mathrm{log}\,{k}_{2}^{T}{e}_{2}$ were conducted at 273.15 K and below-zero temperatures to near the freezing point of the synthetic buffer solutions, as well as at 298.15 K, 283.15 K, and 278.15 K for overlap and direct comparison with the existing data set for purified *m*CP in Liu *et al*.^31^ An estimate of the freezing point of the synthetic buffer solutions was computed from the empirical absolute salinity-temperature relationship of thermally equilibrated sea ice brines^38^, S_A_ = 1000 [1−(54.11/t)]^−1^ where t is the temperature in °C.

## Results and Discussion

### Purification of meta-Cresol Purple

**Figure 2:**
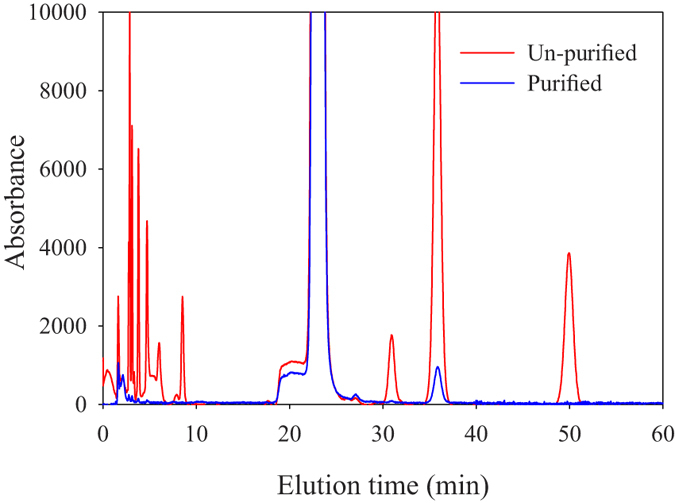
HPLC chromatogram of un-purified (red line) and purified (blue line) *m*CP. Note that traces of a component eluted at approximately 36 minutes are still present in the purified indicator.

Impurities in indicator dyes result in significant uncertainties in measured pH values^31, 39^. Analyses have shown that commercially available *m*CP indicators contain different types and quantities of light absorbing impurities, which could lead to pH offsets as large as 0.01 pH units. Therefore, characterizations of un-purified *m*CP are batch-specific and only valid for pH measurements using the same indicator batch. Measurements generated using uncharacterised un-purified *m*CP can be post-corrected as long as stocks of the un-purified indicator used are archived^31^. The HPLC purification procedure developed by Liu *et al*.^31^ was closely replicated here, yielding approximately 150 mg of purified *m*CP from each injection. Analysis of the un-purified *m*CP indicator following the analytical HPLC protocol of Liu *et al*.^31^ revealed a near identical chromatogram with the exception of an additional peak eluted at about 50 min (Fig. 2). Analysis of the purified material using the same protocol showed complete removal of impurities, with an exception of trace amounts (<8%) of a component eluted at 36 min. Similar residual profiles have been found after purification but have been reported to have practically no effect (<0.001 pH unit) on pH measurements in buffer solutions^40^.

### Molar absorptivity ratios as a function of temperature and salinity

#### e_1_ as a function of temperature

**Figure 3:**
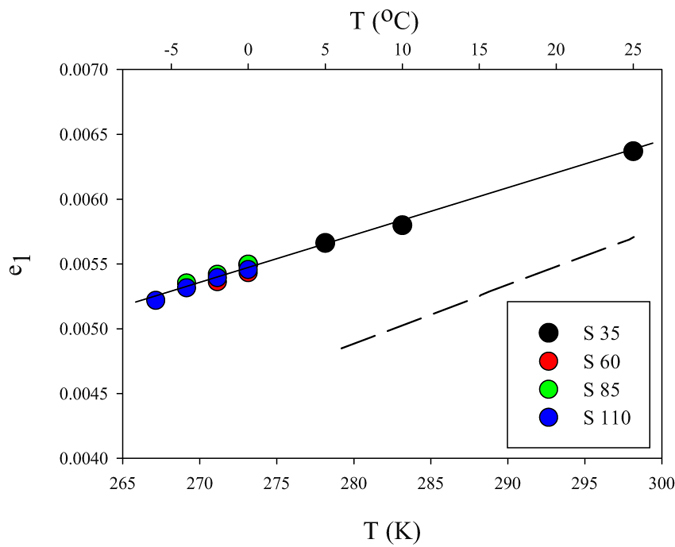
Values of e_1_ as a function of temperature, obtained in NaCl solutions buffered with CH_3_COONa (pH ~4.5) with ionic strengths equivalent to salinities of 35, 60, 85 and 110. The dashed line represents the e_1_ relationship determined by Liu *et al*.^31^.

The temperature dependence of e_1_ for 267.15 K ≤ T ≤ 298.15 K and 35 < S < 110 is relatively small (Fig. 3) and is described by the best-fit equation:

7 $${e}_{1}=-0.004363+3.598\,\times {10}^{-5}{\rm{T}},$$

Although at pH 4.5 the dominant indicator species is HI^−^, small absorbance contributions at 434 and 578 nm from I^2−^ and H_2_I have not been accounted for in our experiments. This may explain why, between 278.15 K and 308.15 K, the best-fit equation (7) above produces e_1_ values between 20% and 10%, respectively, higher than those of Liu *et al*.^31^ (Fig. 3), who found that removing this bias reduced their e_1_ values by a similar magnitude (14–18%). The I^2−^ and H_2_I absorbance contributions are, nonetheless, relatively small, and their effect on pH measurement is minor (<0.0008 pH units) at high R values (>0.7) and slightly larger (up to 0.0034 pH units) at low R values (0.1–0.7)^31^. Refinement of e_1_ to account for the contributions of I^2−^ and H_2_I is possible using an iterative procedure and experimental determinations of ${\varepsilon }_{434}^{{H}_{2}I}$, ${\varepsilon }_{578}^{{H}_{2}I}$, and the K_1_ of *m*CP^31^. This, however, requires careful and laborious experiments offering only minor gain in pH measurement performance especially at pH > 7.5. The potential error in the e_1_ computation from equation (7) above due to the unaccounted absorbance contributions of I^2−^ and H_2_I is not necessarily propagated to the final pH determination (equation 5) but is likely “calibrated out” during the determination of $-\mathrm{log}({k}_{2}^{T}{e}_{2})$ as described subsequently.

Changes in salinity have no significant effect on e_1_ between S = 35 and S = 110 (Fig. 3), consistent with the findings of Liu *et al*.^31^. Generally, e_1_ has a minor influence on the calculation of pH at high pH values (>8). At pH 8, it is possible to disregard the temperature dependence of e_1_ and use an average value with no significant impact on pH (<0.001 pH units) or disregard it altogether (e_1_ = 0) with only a minor effect on pH (0.002 pH units).

#### e_3_/e_2_ as a function of temperature and salinity

**Figure 4:**
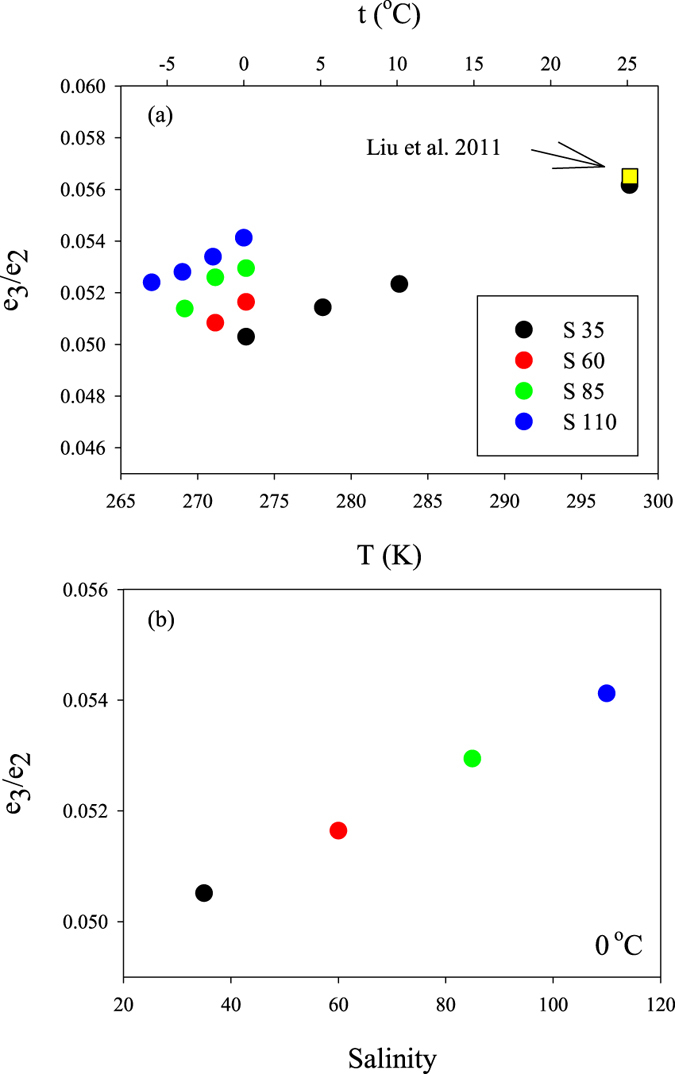
Values of e_3_/e_2_ (**a**) as a function of temperature, and (**b**) salinity at 0 °C. The measurements were obtained at pH 12 in solutions with near-seawater composition and ionic strength equivalent to salinities 35, 60, 85, and 110. The yellow square in panel (a) represents the e_3_/e_2_ value reported by Liu *et al*.^30^ for S = 35 and T = 298.15 K.

The e_3_/e_2_ term in equation 5 is influenced by both the ionic strength and ionic composition^31^ and, for this reason, was determined in an electrolyte solution with near-seawater composition and carefully adjusted ionic strength. The pH was adjusted to ~12 with NaOH so that only the basic (I^2−^) form of *m*CP was present and interferences from HI^−^ and H_2_I were negligible. The temperature and salinity dependence of e_3_/e_2_ (Fig. 4) for 267.15 K < T < 298.15 K and 35 < S < 110 can be described by:

8 $${e}_{3}/{e}_{2}=-0.016224+2.42851\times {10}^{-4}{\rm{T}}+5.05663\times {10}^{-5}(S-35)$$

The relationship provides e_3_/e_2_ values that are in agreement with those reported by Liu *et al*.^31^; at S = 35 and T = 298.15 K, the difference between the values obtained from equation 8 and from the relationship in Liu *et al*.^31^ is 0.0006, which corresponds to a pH discrepancy of less than 0.001 for pH values lower than 8.3. This discrepancy becomes even smaller at lower temperatures. At higher salinities, however, the deviation between the e_3_/e_2_ predicted by the equation of Liu *et al*.^31^ and its value computed from equation 8 above increases to about 0.005, equivalent to ΔpH = 0.010, at S = 100. The expression for e_3_/e_2_ by Liu *et al*.^31^ was optimized for S between 20 and 40, which consequently results in an enhanced discrepancy with our findings at higher salinities. Extrapolation of the Liu *et al*.^31^ e_3_/e_2_ relationship to salinities higher than S = 40 is therefore not advisable. Equation 8 was not experimentally validated at S < 35; nevertheless, it agrees well with that of Liu *et al*.^31^ at S = 20 (the low end of their experimental range), with a maximum discrepancy at 273.15 K of 0.0006 (ΔpH = 0.002).

The pH values obtained using equation 5 are sensitive to variations in e_3_/e_2_ and, therefore, experimental determination requires due care. The multi-point determination of the molar absorptivities of I^2−^ (${\varepsilon }_{434}^{{I}^{2-}}$, ${\varepsilon }_{578}^{{I}^{2-}}$) showed that the intercept of the regression of absorbance *versus* concentration cannot always be assumed as zero. We have observed small but significant intercept offsets in the e_3_/e_2_ determination experiments that, if ignored (e.g., through single point determination), could result in pH errors of ca. 0.001 pH unit. It is not clear what the source of the non-zero intercept is in our experiments, but it may be related to light instabilities of the optical system or other random errors. Benchtop dual-beam spectrophotometers are inherently more stable, allowing for higher quality optical measurements. It is therefore possible that using such instruments eliminates the need for the multi-point determination approach used in this work. This, however, remains to be tested, and it is recommended that, when portable spectrophotometers are used (as in this work), a multi-point determination approach is used.

### Determination of − ${\bf{log}}({{\boldsymbol{k}}}_{{\bf{2}}}^{{\boldsymbol{T}}}{{\boldsymbol{e}}}_{{\bf{2}}})$ as a function of temperature and salinity

The temperature and salinity dependence of $-\mathrm{log}({k}_{2}^{T}{e}_{2})$ of purified *m*CP was determined by measurements of the absorbance ratio (R = A_578_/A_434_) in the Tris/HCl buffers prepared in a range of salinities (S = 35, 45, 50, 60, 70, 85, and 100) at temperatures ranging from their freezing point to 298.15 K. The temperature and salinity dependence of $-\mathrm{log}({k}_{2}^{T}{e}_{2})$ in these conditions can be described by:

9 $$-\mathrm{log}({k}_{2}^{T}{e}_{2})=a+\frac{b}{{\rm{T}}}+c\,\mathrm{ln}\,{\rm{T}}+d{\rm{T}},$$

**Figure 5:**
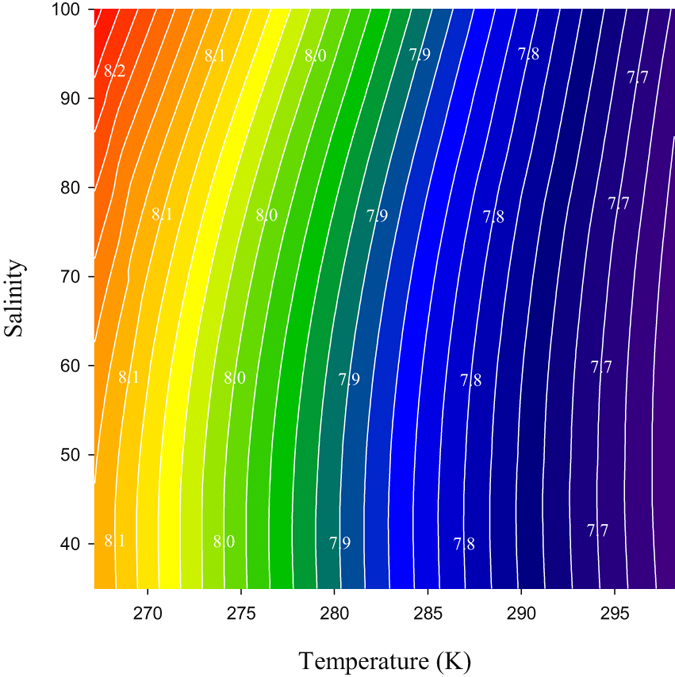
Temperature and salinity dependence of $-\mathrm{log}({{k}}_{2}^{{T}}{{e}}_{2})$ (values on contour lines) as determined in this study from the absorbance ratio (R = A_578_/A_434_) measurements in electrochemically characterized Tris/HCl buffers in synthetic seawater and brines (S = 35, 45, 50, 60, 70, 85, and 100) between their freezing point and 298.15 K.

The factors in the above equation were determined from our measurements using the regression routine in Excel, with a = −319.8369 + 0.688159 *S*−0.00018374 *S* ^2^, b = 10508.724–32.9599 *S* + 0.059082 *S* ^2^, c = 55.54253−0.101639 *S*, d = −0.08112151 (*r* ^2^ = 0.9986, *p* < 0.00001, *n* = 47, standard error of fit: *σ*_*fit*_ = 0.007). Based on this equation, $-\mathrm{log}({k}_{2}^{T}{e}_{2})$ = 8.0171 at 0 °C and S = 35, while $-\mathrm{log}({k}_{2}^{T}{e}_{2})$ = 8.2475 at −6 °C and S = 100. The relatively strong temperature dependence of $-\mathrm{log}({k}_{2}^{T}{e}_{2})$ (Fig. 5) highlights the importance of accurate temperature control (±0.05 °C) during pH measurements. Accurate knowledge of salinity is less important (±1 psu), especially within ranges associated with open ocean waters (30 < S < 40). Under these conditions, salinity variations of the order of 1 psu have only a minor effect on $-\mathrm{log}({k}_{2}^{T}{e}_{2})$ and pH (0.001–0.002 unit) within the uncertainty of the $-\mathrm{log}({k}_{2}^{T}{e}_{2})$ value, based on the standard error of the best-fit S-T function above. At higher salinities (S > 50), more accurate salinity measurements (0.1 psu) are desirable to maintain the same magnitude of $-\mathrm{log}({k}_{2}^{T}{e}_{2})$ and pH uncertainty (in the order of 0.001 pH unit at S = 90).

**Figure 6:**
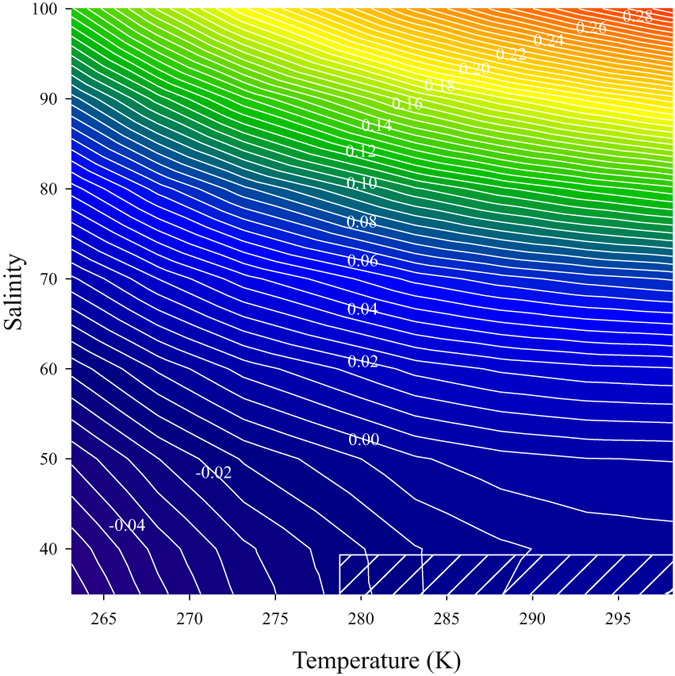
Differences in $-\mathrm{log}({{k}}_{2}^{{T}}{{e}}_{2})$ calculated with the Liu *et al*. (2011) and current parameterizations (equation 8). The hatched rectangle represents a portion of the experimental range of Liu *et al*.^31^.

Liu *et al*.^31^ determined the $-\mathrm{log}({k}_{2}^{T}{e}_{2})$ of purified *m*CP for 278.15 ≤ T ≤ 308.15 and S = 20–40. Our $-\mathrm{log}({k}_{2}^{T}{e}_{2})$S-T parameterization (equation 9) and that in Liu *et al*.^31^ yield values within 0.001 at S = 35 and T = 298.15 ± 5 K and within 0.010 down to T = 283.15 K. Higher discrepancies between the two relationships at low temperatures (Fig. 6) may reflect differences between the instruments used for the $-\mathrm{log}({k}_{2}^{T}{e}_{2})$ determination. The pH measuring system used for this work had no parts of the optical path exposed to air, thus eliminating the possibility of condensation at low temperatures. The condensation is more difficult to control with bench-top spectrophotometers as that used by Liu *et al*.^31^, although dry N_2_ gas was used to eliminate condensation on the optical windows at 5 °C. From this comparison, it is clear that the relationship for $-\mathrm{log}({k}_{2}^{T}{e}_{2})$ by Liu *et al*.^31^ should not be extrapolated for pH measurements outside its range (S = 20–40, T = 278.15–303.15 K) as this can lead to large errors in pH (0.02–0.30) (Fig. 6). The relationship (equation 8) proposed here should also not be used outside its calibration range (S = 35–100, T = 267.15–298.15 K).

### Determination of pH using purified *m*CP at temperatures between 298.15 K and the freezing point of seawater and sea-ice brines up to salinity 100

**Figure 7:**
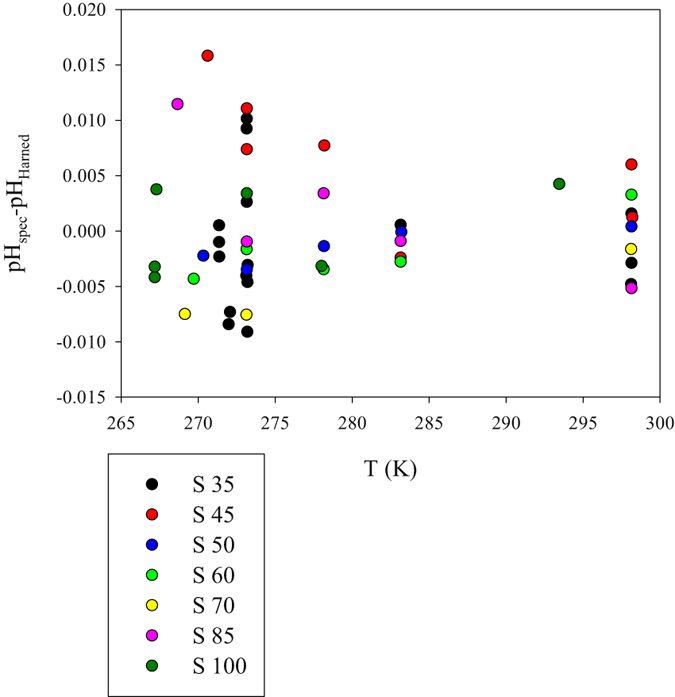
Differences between spectrophotometrically (equations 4, 6, 7 and 8) and electrochemically (Harned) determined pH_T_ in Tris/HCl buffer solutions in synthetic seawater and brines with conservative (seawater-derived) major ionic composition.

Equations 5, 7, 8, and 9 can be used to determine pH on the total proton scale by measurement of the absorption ratio R of purified *m*CP in seawater and seawater brines, with conservative major ionic composition, with S between 30 and 100 and T between freezing point and 298.15 K. The residuals (pH_spec_–pH_Harned_) of pH measurements in Tris/HCl buffers using purified *m*CP and application of eq. 4, 6, 7 and 8 indicate a relatively wide spread (Fig. 7) with an average absolute residual of 0.004 and maximum absolute residual of 0.016. As the analytical precision (1 standard deviation of n = 5–10 repeat measurements of the same buffer) is significantly smaller (0.001–0.004), at least part of the observed magnitude of buffer residuals could be attributed to error propagation from the parameters involved in pH determination (e.g., $-\mathrm{log}({k}_{2}^{T}{e}_{2})$, *σ*_*fit*_ = 0.007) and random error related to buffer preparation, bottling, and handling. Residuals are up to 3 times larger close to the freezing point than at 298.15 K possibly due to the physical/optical heterogeneity of water during the early stages of ice-crystal formation. Therefore, the proposed pH measurement protocol offers good precision (0.001–0.004) and an overall uncertainty in the order of the maximum residual values observed here (0.010–0.020 pH unit), especially at below-zero temperatures near the freezing point of concentrated brines. In comparison, extrapolation of the temperature and salinity dependence of the *m*CP characterization by Liu *et al*.^31^ to values outside their empirical range can lead to pH errors at S = 100 in the order of 0.3 pH unit.

## Summary and Conclusion

We have purified *m*CP and characterized it spectrophotometrically in synthetic solutions with conservative seawater major ionic composition and salinity between 35 and 100 at temperatures ranging from the freezing point of such solutions to 298.15 K. This was made possible by the use of suitable and well characterised Tris/HCl buffers and a novel custom-made optical cell that was fully submerged in a water bath eliminating the possibility of condensation build-up in the optical path. This setup allowed for accurate optical measurements at temperatures down to 267.15 K. Both the experimental set-up and the S-T functions of this work will allow traceable, precise, and reliable spectrophotometric pH measurements in internal sea ice brines and other high latitude and deep waters where temperatures are often just above freezing. The current characterization of purified mCP offers major improvement of pH measurement (0.010–0.020 pH unit uncertainty) in high salinities (up to S = 100) and near-zero and below-zero temperatures to the freezing point over that obtained from the extrapolation of the previous characterization^30^ (0.3 pH unit uncertainty) to these S-T conditions. The important tools developed in this work provide a step forward towards the understanding of the carbonate system in the cryosphere and cold waters in general. In combination with attainable measurements of the remainder three measurable parameters of the carbonate system (DIC, TA, *p*CO_2_), the reliable pH measurements made possible in the extended salinity and temperature ranges of this investigation will facilitate the determination of several unknowns in the parameterization of the carbonate system in these S –T conditions, including the acidity constants of carbonic acid and, following this, important geochemical indicators, such the saturation state of seawater and brines with respect to carbonate minerals in high latitude marine systems.
